# Practical Notes on Diagnosis and Treatment

**Published:** 1912-03-23

**Authors:** 


### PRACTICAL NOTES ON DIAGNOSIS AND TREATMENT.

**Recurrent Ulcer of the Cornea.**

One of the best local applications for this condition is atropine, 1 grain; yellow oxide of mercury (levigated), 2 grains; cocaine, 3 grains; yellow vaseline and lanoline, of each 1 drachm.

**Syphilitic Throat.**

A suitable mouth-wash or gargle for specific ulcers in the buccal cavity, fauces, or pharynx is as follows: Perchloride of mercury, 4 grains; dilute hydrochloric acid, 2 drachms; honey, 2 ounces; water to 8 ounces. At the outset it should be diluted with an equal volume of water.

**Asthma.**

Ten to fifteen drops of pyridine inhaled from a handkerchief will often promptly relieve the paroxysm of asthma. The one drawback to the remedy is its disagreeable and persistent odour. Another method is to put a drachm of pyridine in a saucer in a small room and to keep the patient in the room for fifteen or twenty minutes thrice daily.

**Menstrual Suffering.**

This term is applied by Professor Stephenson, of Aberdeen, to a condition which he believes to be dependent mainly on venous congestion. It is found especially in young women in whom other evidences of sluggish venous circulation are present. Such, for example, are a puffy state of the face, cheeks showing a dull-red flush, and hands cold and bluish red. These are the cases in which potassium permanganate can be administered with success.

**Arsenic and Strychnine.**

These two remedies are often combined, but care is necessary in selecting the appropriate preparations. If Fowler's solution is ordered with liquor strychninæ hydrochlor., the alkali in the former causes a precipitation of strychnine, and hence there is a risk that the last dose contained in the bottle may hold strychnine in excessive quantity. Several cases of poisoning have occurred in this fashion. To avoid this disaster the preparation selected for combination with the strychnine solution should be, not liquor arsenicalis, but liquor arsenic-hydrochloricus.

**Painful Micturition.**

In young children this often means nothing more than an acid condition of the urine. Examination often shows white urates which under the microscope may display "hedgehog" crystals of sodium urate. A few full doses of potassium citrate promptly effects a cure.

**Cannabis Indica in Chorea.**

There are cases of chorea in which cannabis indica acts successfully. Preparations of the drug vary in strength, and care should be taken to use one of standardised and guaranteed efficacy.

**Tachycardia.**

Paroxysmal tachycardia is notoriously resistant to the action of drugs, but it will sometimes yield to repeated small doses of tincture of opium.

**Hæmaturia in Scurvy Rickets.**

Blood in the urine in infants is sometimes the only conspicuous evidence of scurvy rickets. Bleeding from the gums is more common, and there is sometimes epistaxis.

**Stimulants in Pneumonia.**

It is a mistake to give stimulants as a matter of routine. Many patients, especially young patients, do not need them at all. The claim for them is shown by signs of engorgement of the right heart, feeble first sound at the aortic area, and rapid pulse.

**Infantile Diarrhœa.**

Cases of ordinary non-febrile diarrhœa in early life may be successfully treated by oxide of zinc; a dose of 1 to 2 grs. with a few minims of paregoric and a little mucilage and water makes a suitable draught. Thin arrowroot made with equal parts of milk and water is a suitable dietary.

**Thread Worms.**

These are apt to be very persistent in adults. One of the best methods of treatment is thorough evacuation of the colon by purgatives and enemata, followed by slow injection of infusion of quassia so as completely to fill the colon. This may be practised once a week, and a pill of aloes and asafœtida given every night.

**Pruritus Ani.**

In many intractable cases which resist all remedies and systems of dieting the late Sir Mitchell Banks used to apply the actual cautery. He used it freely to the skin around the anus, and never had an unsuccessful case. Occasionally a patient who suffers from pruritus ani gets better when he gives up taking coffee.

**Pyrexia in Cancer.**

The statement is often made that the presence of fever opposes the diagnosis of cancer, say of the liver. This is far too absolute. In many cases there is some fever, and in a few the fever is acute and extreme. In practice the diagnosis is often between hepatic cancer and cirrhosis. Fever suggests the former, but even in alcoholic cirrhosis there are periods of the disease in which the temperature is febrile.

**Sugar-free Milk.**

Milk in cases of diabetes, though an admirable food so far as its protein and fat constituents are concerned, has the disadvantage that it contains sugar. It is worth remembering that sugar-free milk can be obtained, and this is scarcely less palatable than ordinary milk.

